# Skeletal muscle loss is an independent negative prognostic factor in patients with advanced lower rectal cancer treated with neoadjuvant chemoradiotherapy

**DOI:** 10.1371/journal.pone.0195406

**Published:** 2018-04-09

**Authors:** Yasuhiro Takeda, Takashi Akiyoshi, Kiyoshi Matsueda, Hironori Fukuoka, Atsushi Ogura, Hisanori Miki, Yukiharu Hiyoshi, Toshiya Nagasaki, Tsuyoshi Konishi, Yoshiya Fujimoto, Yosuke Fukunaga, Masashi Ueno

**Affiliations:** 1 Gastroenterological Center, Department of Gastroenterological Surgery, Cancer Institute Hospital, Japanese Foundation for Cancer Research, Tokyo, Japan; 2 Department of Diagnostic Imaging, Cancer Institute Hospital, Japanese Foundation for Cancer Research, Tokyo, Japan; Universidade de Mogi das Cruzes, BRAZIL

## Abstract

**Background:**

The impact of body composition on the short- or long-term outcomes of patients with surgically treated advanced rectal cancer after neoadjuvant chemoradiotherapy remains unclear. This study examined the correlation between low skeletal muscle mass and morbidity and survival in patients with advanced lower rectal cancer.

**Methods:**

We enrolled 144 clinical stage II/III patients with advanced lower rectal cancer who underwent neoadjuvant chemoradiotherapy followed by curative resection between 2004 and 2011. The cross-sectional skeletal muscle area at the third lumbar vertebra (L3) level was evaluated by computed tomography before chemoradiotherapy, and this was normalized by the square of the height to obtain the skeletal muscle index. Low skeletal muscle mass was defined as the sex-specific lowest quartile of the L3 skeletal muscle index. The association between low skeletal muscle mass and morbidity, relapse-free survival, or overall survival was assessed.

**Results:**

Low skeletal muscle mass was identified in 37 (25.7%) patients. Age and body mass index were associated with low skeletal muscle mass. By multivariate analysis, we found that low skeletal muscle mass was independently associated with poor overall survival (hazard ratio = 2.93; 95%CI: 1.11–7.71; p = 0.031) and relapse-free survival (hazard ratio = 2.15; 95%CI: 1.06–4.21; p = 0.035), but was not associated with the rate of postoperative complications.

**Conclusions:**

Low skeletal muscle mass is an independent negative prognostic factor for relapse-free and overall survival in patients with advanced lower rectal cancer treated with neoadjuvant chemoradiotherapy.

## Introduction

Neoadjuvant chemoradiotherapy (NCRT) is the standard of care for patients with advanced lower rectal cancer because of the relatively high risk of local recurrence. However, NCRT is also associated with a higher toxicity than surgery alone, including increased postoperative complications or long-term bowel and urogenital dysfunction [1, 2]. Therefore, it is becoming increasingly important to identify factors that can predict patient responses in terms of morbidity and survival to select those who could most benefit from NCRT.

Sarcopenia, initially defined as an age-related decrease in muscle mass and strength, is associated with chronic disease states, including various malignancies [3, 4]. Its etiology is multifactorial, and leads to nutritional changes, inflammation, and altered endocrine function [5]. Skeletal muscle loss is the pivotal feature of sarcopenia, and it is associated with chemotherapy toxicity, complications following surgery, and a decreased rate of survival in cancer patients [6–12]. Thus, it is worth considering whether skeletal muscle loss could be used to predict morbidity and survival among patients with advanced lower rectal cancer who are treated with NCRT.

Although muscle mass has been traditionally assessed by bioelectrical impedance analysis (BIA) or dual-energy X-ray absorptiometry (DXA), there has been a recent shift to using computed tomography (CT) and magnetic resonance imaging (MRI) for measurements [13]. Indeed, CT is routinely performed to assess for primary lesions and to detect distant metastases in patients with advanced rectal cancer. In the present study, we hypothesized that reduced muscle mass before NCRT evaluated by preoperative CT might be associated with higher morbidity or lower survival rates in patients with advanced lower rectal cancer scheduled for NCRT. The main finding of this study is that low skeletal muscle mass is an independent negative prognostic factor for relapse-free and overall survival in patients with advanced lower rectal cancer scheduled for NCRT.

## Materials and methods

### Patients

**Fig 1:**
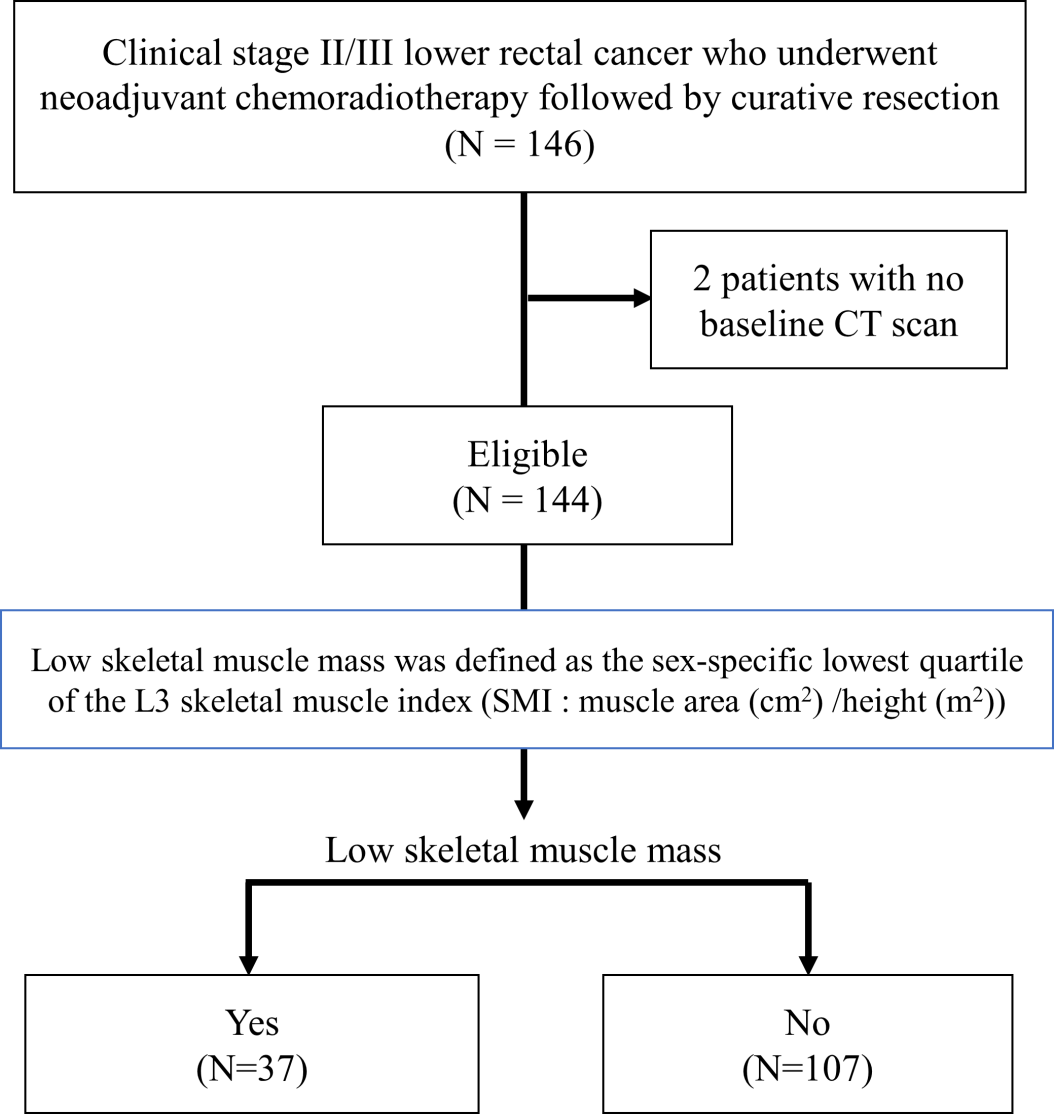
Flow chart of the study.

This retrospective study was approved by the institutional review board of the Cancer Institute Hospital (Ariake, Tokyo; approval number “2016–1184”). Written informed consent was waived because of the retrospective design. From July 2004 to December 2011, 146 patients with clinical stage II/III advanced lower rectal cancer in which the inferior border was located below the peritoneal reflection underwent long-course NCRT (45–50.4 Gy with oral 5-fluorouracil chemotherapy) and curative resection at our hospital. Preoperative tumor staging before and after CRT was performed by physical examination, colonoscopy, CT, and MRI. Total mesorectal excision was performed 4 to 8 weeks after the completion of NCRT. Among the 146 patients, pre-treatment abdominal CT images were not available for 2 patients because imaging was performed in another hospital; therefore, the final cohort comprised 144 patients (Fig 1). All data were fully anonymized before the analysis.

### Clinico-pathological data

Clinico-pathological data were collected, including sex, age, body mass index (BMI), carcinoembryonic antigen (CEA) level, carbohydrate antigen (CA) 19–9 level, tumor distance from the anal verge, clinical staging, operative details, postoperative complications, pathology, and follow-up data. Physical examination, colonoscopy, CT, and MRI were performed before and after NCRT. Complications were scored according to the Clavien-Dindo classification [14], with major complications defined as having a Clavien-Dindo grade above IIIa. Pathological examinations were carried out according to the Union for International Cancer Control TNM staging system. Tumor regression was graded according to the definition proposed by Dworak [15].

### CT analysis and evaluation of low skeletal muscle mass

Skeletal muscle mass was assessed by a retrospective evaluation of contrast-enhanced CT scans obtained before the start of NCRT. The three-dimensional image analysis system (SYNAPSE VINCENT; Fujifilm Medical, Tokyo, Japan) used specific tissue demarcations to delineate the tissues of interest, and then took semi-automated measurements of cross-sectional skeletal muscle areas at the level of L3 (cm^2^) within a Hounsfield unit (HU) threshold range of −30 to +150 for skeletal muscle. The most caudal images were selected to measure the skeletal muscle areas, including the psoas muscles, paraspinous muscles (quadratus lumborum, erector spinae) and abdominal wall muscles [9, 16]. These values were normalized against the square of the patient’s height (m^2^) to obtain the skeletal muscle index (SMI, cm^2^/m^2^). Patients were stratified by quartiles according to the SMI values for men and women. Low skeletal muscle mass was defined as the lowest quartile [9, 16].

### Postoperative follow-up

Postoperative physical examinations and laboratory blood tests were conducted. CEA and CA19-9 levels were measured every 3 months during the first 3 years, every 6 months over the next 2 years, then annually. CT evaluations were performed every 6 months according to the Japanese Society for the Cancer of the Colon and Rectum guidelines [17]. Colonoscopy was performed one year after surgery, and then performed every 2 years.

### Statistical analysis

Associations between the SMI and clinico-pathological characteristics were investigated using Fisher’s exact test for categorical variables and Mann–Whitney *U*-test for continuous variables. Overall survival (OS) was defined from the date of surgery to the date of death, and relapse-free survival (RFS) from the date of surgery to the date of rectal cancer recurrence. OS and RFS curves were evaluated by the Kaplan–Meier method using the log-rank test. To identify potential predictors of OS and RFS, a univariate Cox proportional hazard model was used to compute the hazard ratio (HR) and 95% confidence interval (CI). After univariate analysis, variables with a *P* value < 0.10 were selected for multivariate analysis. A *P* value < 0.05 was considered to indicate statistical significance. Statistical analyses were performed using JMP 13 (SAS Institute Inc. Cary, NC, USA).

## Results

The detailed clinico-pathological characteristics of all 144 patients are provided in S1 Table. There were 102 (70.8%) males and 42 (29.2%) females, with a median BMI of 23.0 kg/m^2^ (range, 16.2–39.5 kg/m^2^). Sixty patients (41.7%) were diagnosed with pathological stage III disease.

The median SMI was 46.2 cm^2^/m^2^. The SMI values were significantly lower in females than in males (37.72 cm^2^/m^2^ vs. 48.48 cm^2^/m^2^, *P* < 0.001). The lowest quartile SMI threshold was 45.0 cm^2^/m^2^ for males and 33.8 cm^2^/m^2^ for females. Thus, 37 (25.7%) patients were diagnosed with low skeletal muscle mass.

Patients with low skeletal muscle mass were significantly older and had a significantly lower BMI (Table 1). There were no other significant associations between low skeletal muscle mass and clinicopathological characteristics.

**Table 1:** Clinico-pathological characteristics of the patients.

Variable	Low Skeletal Muscle Mass		*P* value
	Yes (n = 37)	No (n = 107)	
Sex, male (%)	26 (70.3)	76 (71.0)	1
Median age (years, range)	65 (42–81)	60 (32–75)	0.009
Median BMI (kg/m^2^, range)	20.6 (16.0–27.9)	23.9 (16.9–39.5)	<0.001
Tumor distance from AV (mm)	30 (10–80)	40 (10–80)	0.113
Pretreatment CEA ≥ 5 (ng/ml)	15 (40.5)	42 (39.3)	1
Pretreatment CA19-9 ≥ 37 (ng/ml)	5 (13.5)	13 (12.2)	0.780
Cell differentiation other than well/moderate (%)	5 (13.5)	10 (9.4)	0.534
Clinical stage III (%)	23 (62.2)	76 (71.0)	0.411
Laparoscopic surgery (%)	29 (78.4)	82 (76.6)	1
Operating time ≥ 360 min (%)	8 (21.6)	32 (29.9)	0.398
Estimated blood loss ≥100 ml (%)	17 (46.0)	46 (43.0)	0.848
Postoperative complications (%)	9 (24.3)	32 (29.9)	0.673
Postoperative complication ≥ IIIa (%)	1 (2.7)	9 (8.4)	0.453
Tumor regression grade ≥ 3 (%)	8 (21.6)	20 (18.7)	0.891
Pathological stage III (%)	14 (37.8)	46 (43.0)	0.700
Adjuvant chemotherapy (%)	13 (35.1)	50 (46.7)	0.252

*BMI* body mass index, *AV* anal verge, *CEA* carcinoembryonic antigen, *CA19-9* carbohydrate antigen 19–9

Postoperative complications occurred in 41 patients (28.5%), among which 10 (6.9%) had Clavien–Dindo grade ≥ IIIa complications. Only 1 of these 10 patients had low skeletal muscle mass, and this patient underwent re-operation due to anastomotic leakage (grade IIIb). The complications in the other 9 patients without low skeletal muscle mass were anastomotic leakage (*n* = 3, grade IIIa in 2; grade IIIb in 1), surgical site infection (*n* = 4, grade IIIa), ileus (*n* = 1, grade IIIa), and stroke (*n* = 1, grade IVa). There was no mortality. Low skeletal muscle mass was not significantly associated with the incidence of overall (p = 0.673) or major (p = 0.453) complications.

**Fig 2:**
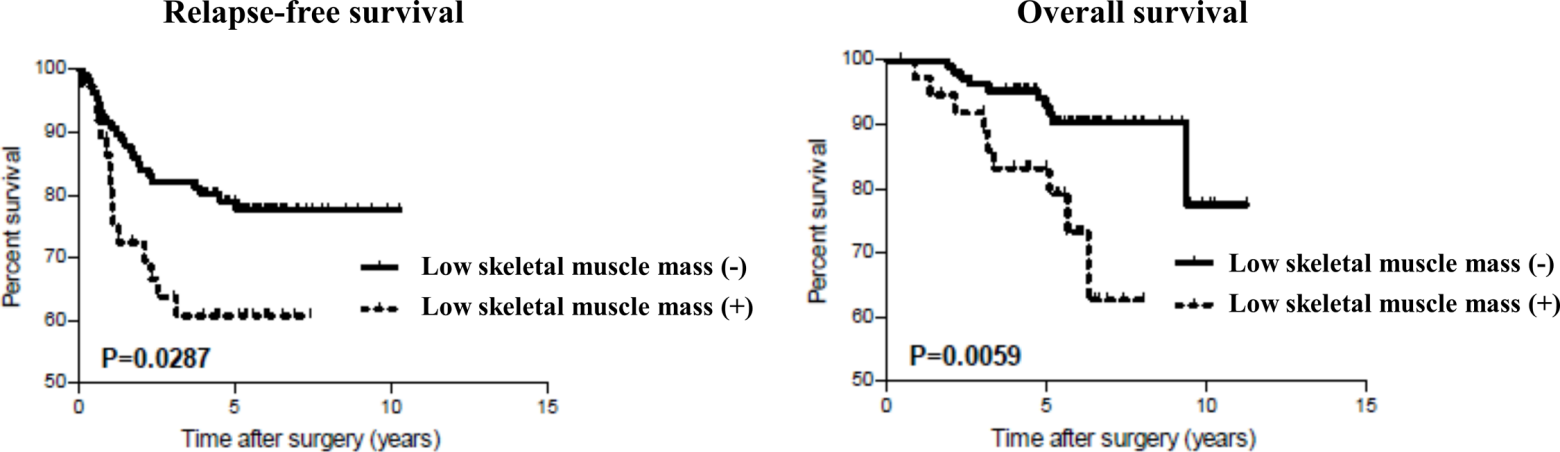
Kaplan-Meier curves for relapse-free survival and overall survival after surgery.

The median follow-up was 67.6 months (range, 5.7 to 137.1 months), during which time there were 37 recurrences (25.7%) and 19 deaths (13.2%). Patients with low skeletal muscle mass had a significantly poorer RFS (5-year RFS, 79.1% vs. 60.7%, *P* = 0.029) and OS (5-year OS, 93% vs. 83.2%, *P* = 0.006; Fig 2) than those with normal skeletal muscle mass. In the univariate analysis, both RFS and OS were significantly associated with low skeletal muscle mass, tumor cell differentiation, and pathological stage. In the multivariate analysis, low skeletal muscle mass and pathological stage were independently associated with poor RFS (HR [95%CI], 2.15 [1.06 to 4.21], *P* = 0.035 and 3.78 [1.85 to 8.26], *P* < 0.001, respectively; Table 2) and poor OS (2.93 [1.11 to 7.71]; *P* = 0.031 and 3.31 [1.27 to 9.60], *P* = 0.014, respectively; Table 3).

**Table 2:** Univariate and multivariate analyses of factors associated with relapse-free survival in patients with advanced lower rectal cancer undergoing curative resection.

Variable	Univariate	*P* value	Multivariate	*P* value
	HR (95%CI)		HR (95%CI)	
Sex (male)	0.851 (0.437–1.755)	0.646		
Age > 60 years	1.560 (0.812–3.107)	0.186		
BMI ≥ 25 kg/m^2^	1.163 (0.551–2.293)	0.674		
Low skeletal muscle mass	2.069 (1.038–3.981)	0.029	2.148 (1.058–4.208)	0.035
Tumor distance from AV < 50 mm	1.088 (0.566–2.167)	0.804		
Pretreatment CEA ≥ 5 ng/ml	1.150 (0.591–2.195)	0.674		
Pretreatment CA19-9 ≥ 37 ng/ml	2.038 (0.868–4.251)	0.069	1.422(0.597–3.024)	0.404
Differentiation (well/moderate)	0.439 (0.204–1.089)	0.044	1.828 (0.721–4.041)	0.189
Clinical stage III	1.483 (0.728–3.333)	0.301		
Laparoscopic surgery	0.654 (0.332–1.380)	0.234		
Operating time ≥ 360 min	1.456 (0.720–2.814)	0.272		
Estimated blood loss ≥ 100 ml	1.213 (0.632–2.320)	0.557		
Any postoperative complications	1.622 (0.814–3.115)	0.150		
Postoperative complications ≥ IIIa	1.697 (0.506–4.271)	0.312		
Tumor regression grade ≥ 3	0.328 (0.079–0.913)	0.052	1.834 (0.625–7.833)	0.295
Pathological stage III	4.261 (2.157–9.006)	<0.001	3.778 (1.853–8.259)	<0.001
Adjuvant chemotherapy	1.277 (0.665–2.443)	0.457		

*HR* hazard ratio, *CI* confidence interval, *BMI* body mass index, *AV* anal verge, *CEA* carcinoembryonic antigen, *CA19-9* carbohydrate antigen 19–9.

**Table 3:** Univariate and multivariate analyses of factors associated with overall survival in patients with advanced lower rectal cancer undergoing curative resection.

Variable	Univariate	*P* Value	Multivariate	*P* Value
	HR (95%CI)		HR (95%CI)	
Sex (male)	0.630 (0.253–1.694)	0.327		
Age > 60 years	1.990 (0.786–5.664)	0.155		
BMI ≥ 25 kg/m^2^	0.750 (0.212–2.091)	0.610		
Low skeletal muscle mass	3.395 (1.324–8.707)	0.006	2.932 (1.105–7.705)	0.031
Tumor distance from AV < 50 mm	1.406 (0.554–4.009)	0.489		
Pretreatment CEA ≥ 5 ng/ml	1.999 (0.808–5.168)	0.129		
Pretreatment CA19-9 ≥ 37 ng/ml	2.562 (0.827–6.707)	0.061	1.728 (0.528–4.780)	0.341
Differentiation (well/moderate)	0.332 (0.124–1.060)	0.032	2.260 (0.688–6.315)	0.166
Clinical stage III	1.364 (0.521–4.228)	0.550		
Laparoscopic surgery	0.589 (0.233–1.611)	0.270		
Operating time ≥ 360 min	1.181 (0.415–2.989)	0.736		
Estimated blood loss ≥ 100 ml	1.602 (0.642–4.167)	0.311		
Any postoperative complications	0.728 (0.222–1.978)	0.559		
Postoperative complications ≥ IIIa	1.260 (0.196–4.510)	0.760		
Tumor regression grade ≥ 3	0.366 (0.055–1.359)	0.184		
Pathological stage III	3.618 (1.425–10.323)	0.006	3.306 (1.271–9.597)	0.014
Adjuvant chemotherapy	1.388 (0.540–3.567)	0.487		

*HR* hazard ratio, *CI* confidence interval, *BMI* body mass index, *AV* anal verge, *CEA* carcinoembryonic antigen, *CA19-9* carbohydrate antigen 19–9.

## Discussion

Initially proposed as an age-related muscle mass decrease [3], sarcopenia is currently classified by the presence of both low muscle mass and low muscle function, according to the current definition of The European Working Group on Sarcopenia in Older People, with patients at risk of adverse outcomes such as poor quality of life, physical disability, and death [13]. Some studies have shown that sarcopenia is also associated with negative clinical outcomes in patients with malignant diseases; although, many studies in cancer patients evaluated only skeletal muscle depletion without evaluating skeletal muscle strength [6–12] [18–23]. Few studies have examined the impact of sarcopenia in patients with advanced lower rectal cancer without distant metastasis who are undergoing NCRT. In the present study, we showed that low skeletal muscle mass is independently associated with poor OS and poor RFS in patients with advanced lower rectal cancer, despite the absence of a significant association with postoperative complications.

Several previous studies have shown that low skeletal muscle mass predicts survival in cancer patients [9, 18, 19]. Malietzis et al. [19] and Miyamoto et al. [9], as both groups showed that low skeletal muscle mass defined based on the skeletal muscle area at L3 on CT was a poor prognostic factor in patients who underwent surgery for colorectal cancer. However, these studies included patients with colon and rectal cancer, and those classified with stage I thorough stage IV disease. We included only patients with clinical stage II/III advanced lower rectal cancer below the peritoneal reflection who were undergoing NCRT. The results showed that low skeletal muscle mass is an independent negative predictive factor of survival in this group of patients. A recent report [20] also showed that low skeletal muscle mass was negatively associated with OS in patients with advanced rectal cancer treated with NCRT, although the cut-off value to define low skeletal muscle mass was different from that in our study.

In the present study, we found no association between skeletal muscle mass and postoperative complications; yet, several other studies have indicated an association between skeletal muscle mass and short-term outcomes in colorectal cancer or colorectal liver metastases [8, 10, 21, 22]. Peng and colleagues showed that sarcopenia defined based on the total psoas muscle area on CT increased the risk of postoperative complications in patients with colorectal liver metastasis, which led to an extended stay in the intensive care unit after surgery, and a longer hospital stay [10]. Lieffers and others also showed that sarcopenia similarly only defined based on the skeletal muscle area at L3 on CT was associated with an increased risk of perioperative infection, a longer hospital stay, and prolonged rehabilitation care in patients who underwent resection of colorectal cancer [8]. One possible explanation for this inconsistencies between our study and others is that there is no standardized cutoff value for defining low skeletal muscle mass by CT, and this has resulted in varying definitions across a range of studies [9–11, 18, 23, 24]. The most commonly used definition of low skeletal muscle mass was proposed by Prado et al. [11] and this relies on the SMI, with a cutoff value of 52.4 cm^2^/m^2^ for males and 38.5 cm^2^/m^2^ for females. If we had applied this definition in our study, then as many as 101 (70.1%) patients would have been classified as having low skeletal muscle mass, as compared with 37 (25.7%) using our cut-off values; this better reflects the rates of patients with low skeletal mass in the studies of Peng et al. (15.8%) [10] and Lieffers et al. (38.9%) [8]. Moreover, the cutoff value that defines sarcopenia differs between populations [25], and the CT-based values relevant for Asian populations remains unclear. In this study, low skeletal muscle mass was defined using a sex-specific cutoff below the lowest quartile. This definition of low skeletal muscle mass has been applied in several other studies in which a relationship with postoperative outcome or prognosis has been demonstrated [9, 23, 26, 27].

The reasons why low skeletal muscle mass is negatively associated with OS and RFS in patients with advanced rectal cancer treated with NCRT are likely to be multifactorial. Previous studies have shown a significant association between low skeletal muscle mass and host systemic inflammatory response in patients with colorectal cancer [28, 29]. Local and systemic inflammation are poor prognostic factors in patients with rectal cancer treated with NCRT [30, 31]. Skeletal muscle wasting involves the production of pro-inflammatory cytokines derived from tumor and immune cells, and negative regulation of insulin-like growth factor-1 signaling by myostatin and activin [32]. Further study is needed to uncover the potential molecular mechanisms driving muscle loss.

In the present study, low skeletal muscle mass and pathological stage were independently associated with RFS and OS. Of these, only low skeletal muscle mass can act as a prognostic factor before treatment. If low muscle mass has a direct link with survival, it is possible that endurance or resistance exercise training and increased physical activity might be able to modify muscle mass before surgery [33]. Alternatively, nutritional intervention, including increased protein intake, might be a promising approach to counteract the anabolic resistance associated with inactivity or inflammation [34]. Either or both approaches may be feasible during the 3 to 4 months that typically precede surgery in patients receiving preoperative CRT for advanced rectal cancer. Indeed, recent clinical and preclinical studies have shown that multimodal interventions, including nutritional support, physical exercise, and pharmacological intervention, are necessary to treat patients with muscle wasting and cancer cachexia [35, 36]. Further studies are needed to confirm how useful these interventions will be in improving patient outcomes.

The present study had several limitations. First, it was a retrospective, single-institution analysis; multi-institutional, prospective validation study is necessary to confirm our results. Second, although The European Working Group on Sarcopenia in Older People defines sarcopenia as the presence of both low muscle mass and low muscle strength or low physical performance, muscle function and strength could not be measured because of the retrospective study design. However, most of the previous studies evaluating the association between sarcopenia and outcomes in cancer patients diagnosed sarcopenia only by low skeletal muscle mass using CT. Third, CT scans were obtained at the time of diagnosis, and we cannot know the changes in muscle mass and ongoing weight loss that occurred before diagnosis. This is a key criteria of cancer cachexia and it is therefore unclear how many patients with low skeletal muscle mass in the present study can be classified as having cancer cachexia. Finally, as mentioned above, there is no consensus regarding the definition of sarcopenia, nor are there standardized, CT-defined cutoff values for its measurement. These are important considerations that need to be addressed in future studies.

## Conclusion

In conclusion, the present study shows that low skeletal muscle mass, an important feature of sarcopenia, is independently associated with poor OS and poor RFS in patients with advanced lower rectal cancer undergoing NCRT, despite the absence of a significant association with postoperative complications.

## Supporting information

S1 Table Clinicopathological characteristics. (XLS)
